# Supplementary material for: Direct detection of electrogenic polyamine transport
Source: bioRxiv. 2026 Oct 7:2026.10.01.755726. Preprint. [Version 1] doi: 10.64898/2026.10.01.755726 (PMC13691954; doi:10.64898/2026.10.01.755726)
Supplement: Supplementary file 1 [file media-1.pdf]

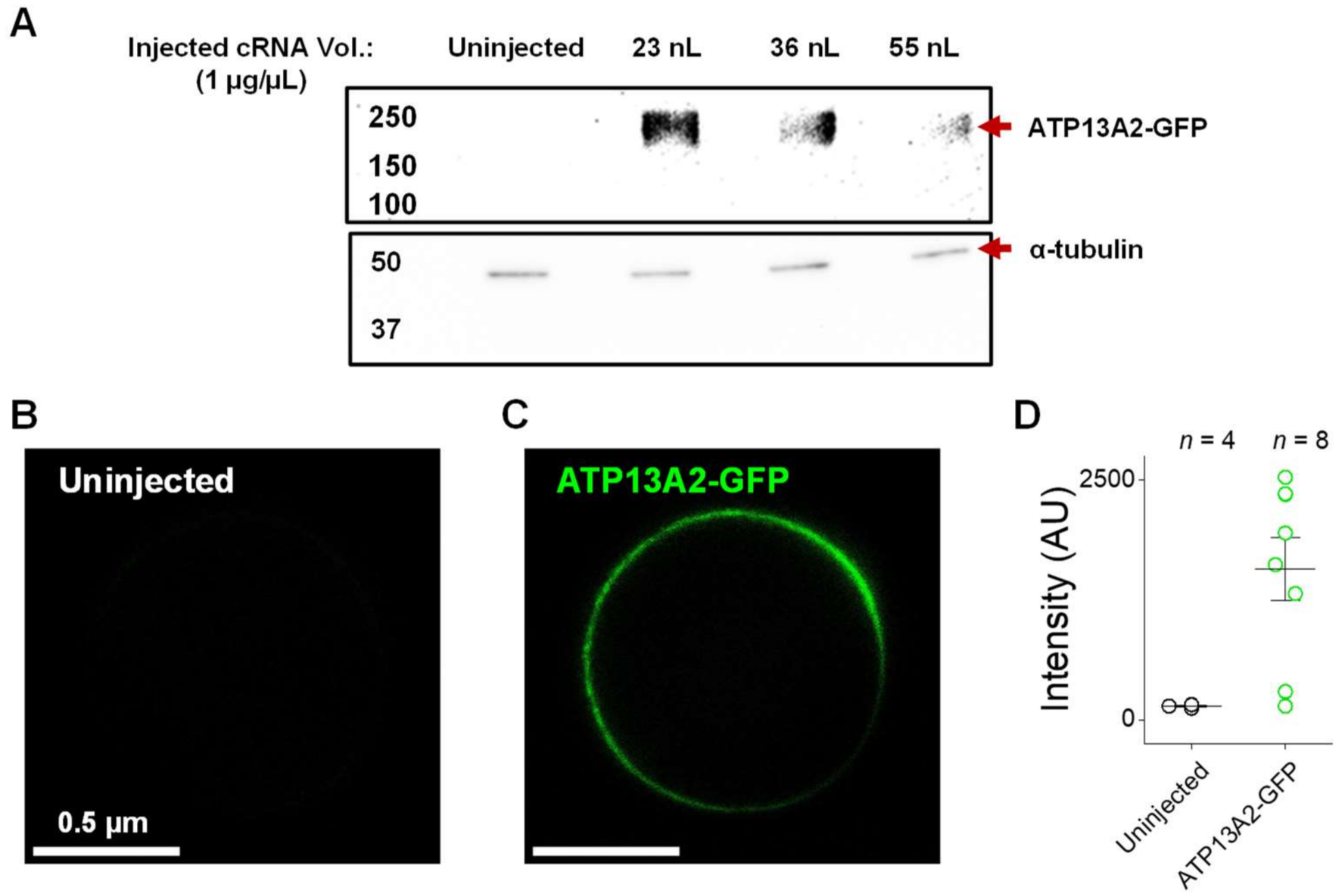

Supplementary Figure S1. A) Western blot of lysates from oocytes injected with ATP13A2-GFP cRNA immunostained with anti-ATP13A2 and -alpha-tubulin antibodies, shows that injecting lower volumes of cRNA (1 µg/µL) resulted in higher ATP13A2 expression. **B, C)** Confocal image of uninjected (*B*) or ATP13A2-GFP injected (*C*) oocytes taken with a 10X objective. **D)** Quantification of GFP fluorescence intensity of uninjected oocytes and oocytes injected with ATP13A2-GFP cRNA.
