# Supplementary material for: Direct detection of electrogenic polyamine transport
Source: bioRxiv. 2026 Oct 7:2026.10.01.755726. Preprint. [Version 1] doi: 10.64898/2026.10.01.755726 (PMC13691954; doi:10.64898/2026.10.01.755726)
Supplement: Supplementary file 3 [file media-3.pdf]

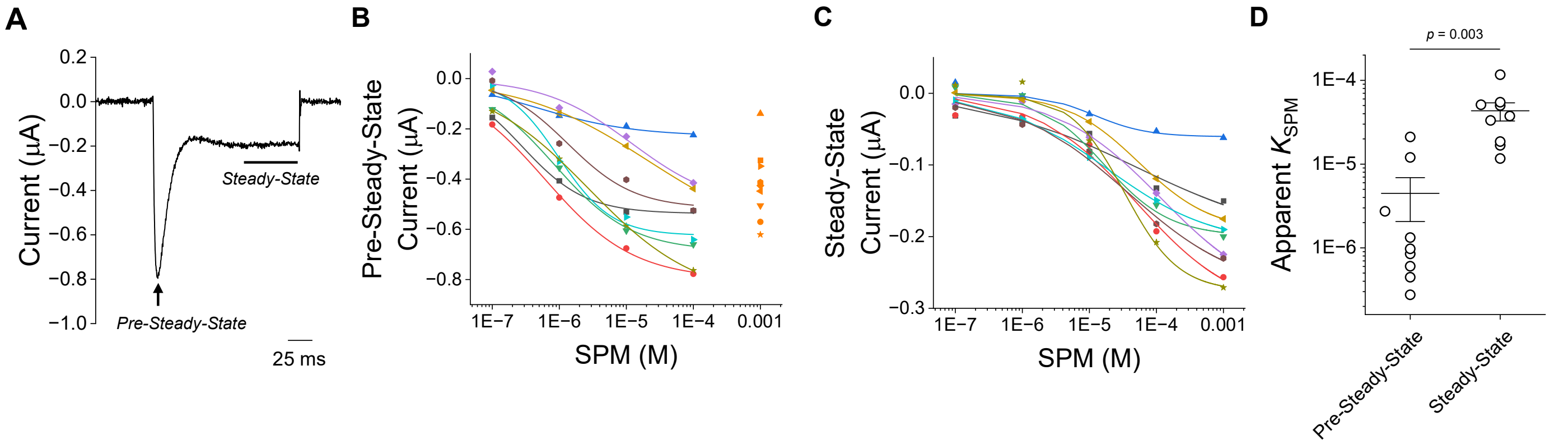

Supplementary Figure S3. A) Representative SPM-induced currents elicited from ATP13A2 injected oocytes during a 150 ms voltage step from 0 to -190 mV. **B, C)** Dose-dependent activation pre-steady-state (*B*) and steady-state currents (*C*) (n = 9). Data from each independent oocyte were fit with a Hill equation. For the pre-steady-state component, only the ascending phase was fit with Hill equation. The orange points at high SPM concentration were therefore excluded from the fits. **D)** Apparent K_{SPM} was significantly lower for the pre-steady-state component compared to the steady-state (p = 0.004, two-tailed paired t-test).
