# Supplementary material for: Direct detection of electrogenic polyamine transport
Source: bioRxiv. 2026 Oct 7:2026.10.01.755726. Preprint. [Version 1] doi: 10.64898/2026.10.01.755726 (PMC13691954; doi:10.64898/2026.10.01.755726)
Supplement: Supplementary file 4 [file media-4.pdf]

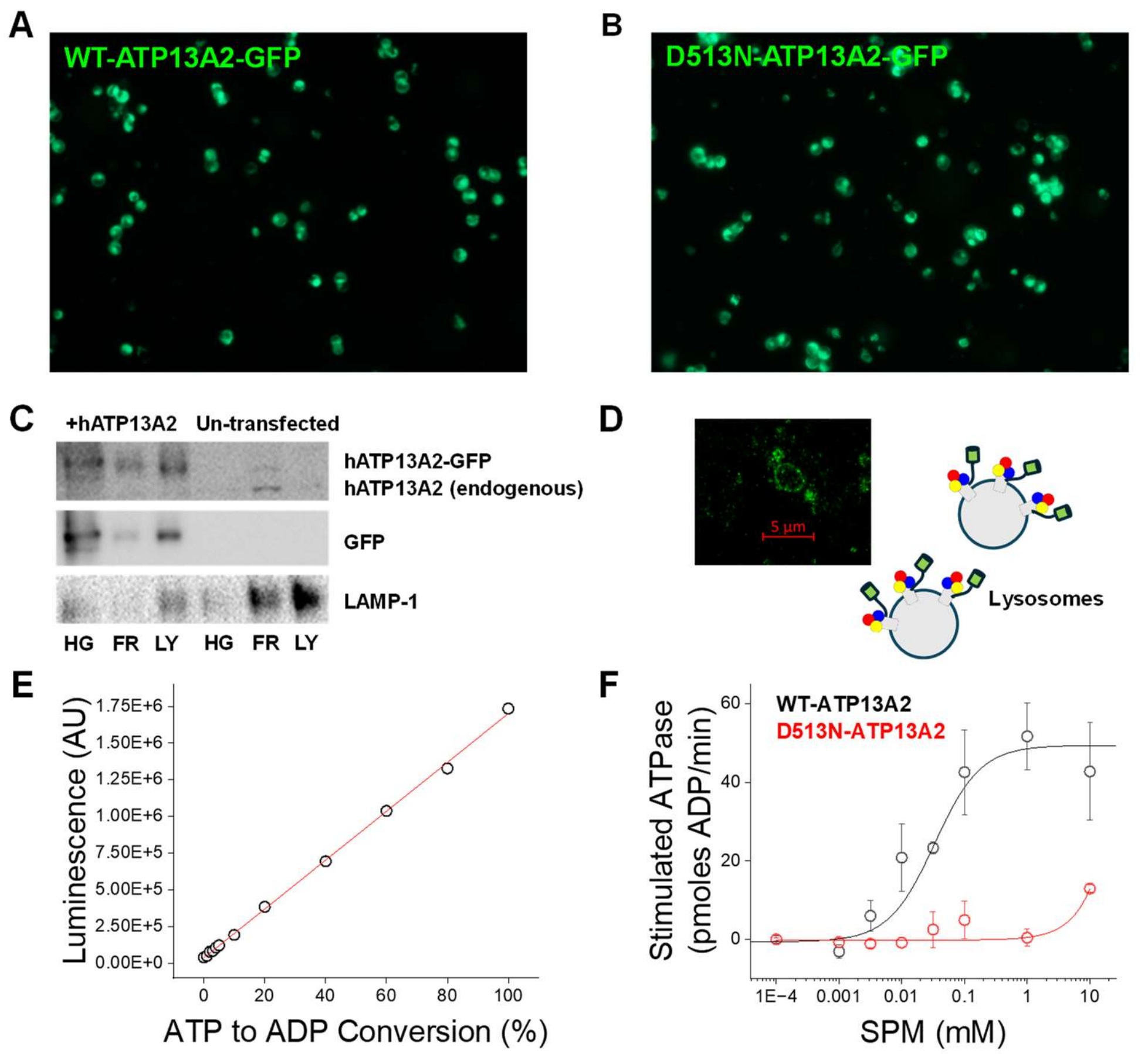

Supplementary Figure S4. A,B) HEK293S cells expressing WT- (A) or D513N- (B) ATP13A2-GFP after baculoviral transduction. **C)** Western blot demonstrates that ATP13A2-GFP and LAMP-1 are co-enriched as the samples are purified from the starting cellular homogenates (HG) to organellar membrane fractions (FR), and to lysosomal fractions (LY). **D)** Confocal image of lysosomes after they were enlarged by pre-treating HEK293 cells with 1 μM vacuolin and isolated through homogenization and centrifugation. **E)** A standard curve was used to estimate ATP to ADP conversion based on ATP-dependent activation of luciferase luminescence. **F)** Stimulated ATPase activity of lysosomal fractions from cells expressing WT-ATP13A2-GFP (black) or D513N-ATP13A2-GFP (red). Data are mean ± SEM (n = 3). Stimulated ATPase activity from WT-ATP13A2 was well described by a Hill equation (apparent K_m = 33.4 ± 12.7 μM, *Hill coefficient* = 1.2 ± 0.6, R^2 = 0.971).
