# Supplementary material for: Direct detection of electrogenic polyamine transport
Source: bioRxiv. 2026 Oct 7:2026.10.01.755726. Preprint. [Version 1] doi: 10.64898/2026.10.01.755726 (PMC13691954; doi:10.64898/2026.10.01.755726)
Supplement: Supplementary file 5 [file media-5.pdf]

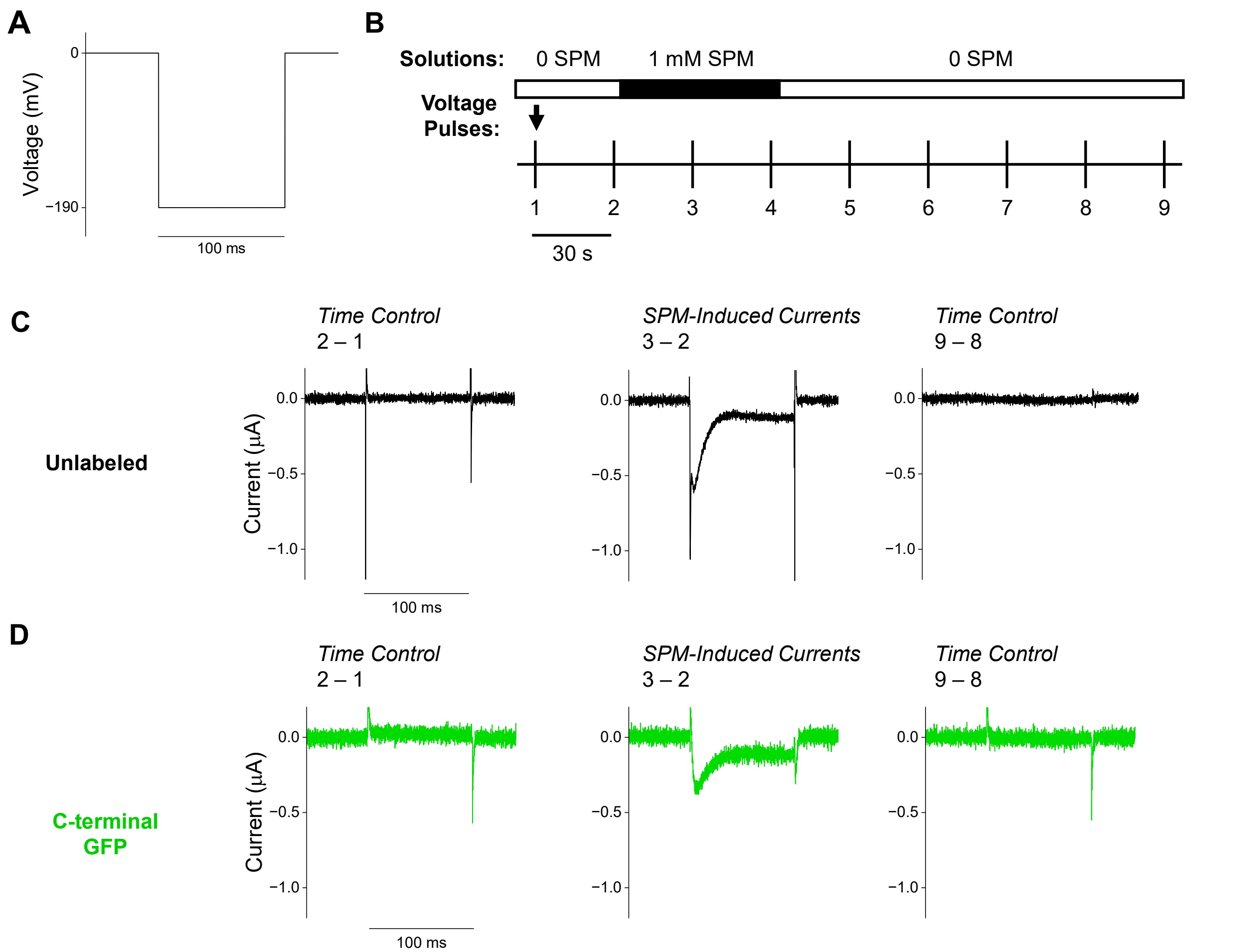

Supplementary Figure S5. A, B) Voltage steps from 0 to -190 mV (100 ms pulse, holding potential = 0 mV) were applied to oocytes every 30 seconds while perfusing with bath solution ± 1 mM SPM. **C,D)** Similar currents were elicited from oocytes injected with unlabeled- (*C*) or C-terminal GFP-labeled- ATP13A2 cRNA (*D*) within the same batch.
