# Supplementary material for: Direct detection of electrogenic polyamine transport
Source: bioRxiv. 2026 Oct 7:2026.10.01.755726. Preprint. [Version 1] doi: 10.64898/2026.10.01.755726 (PMC13691954; doi:10.64898/2026.10.01.755726)
Supplement: Supplementary file 6 [file media-6.pdf]

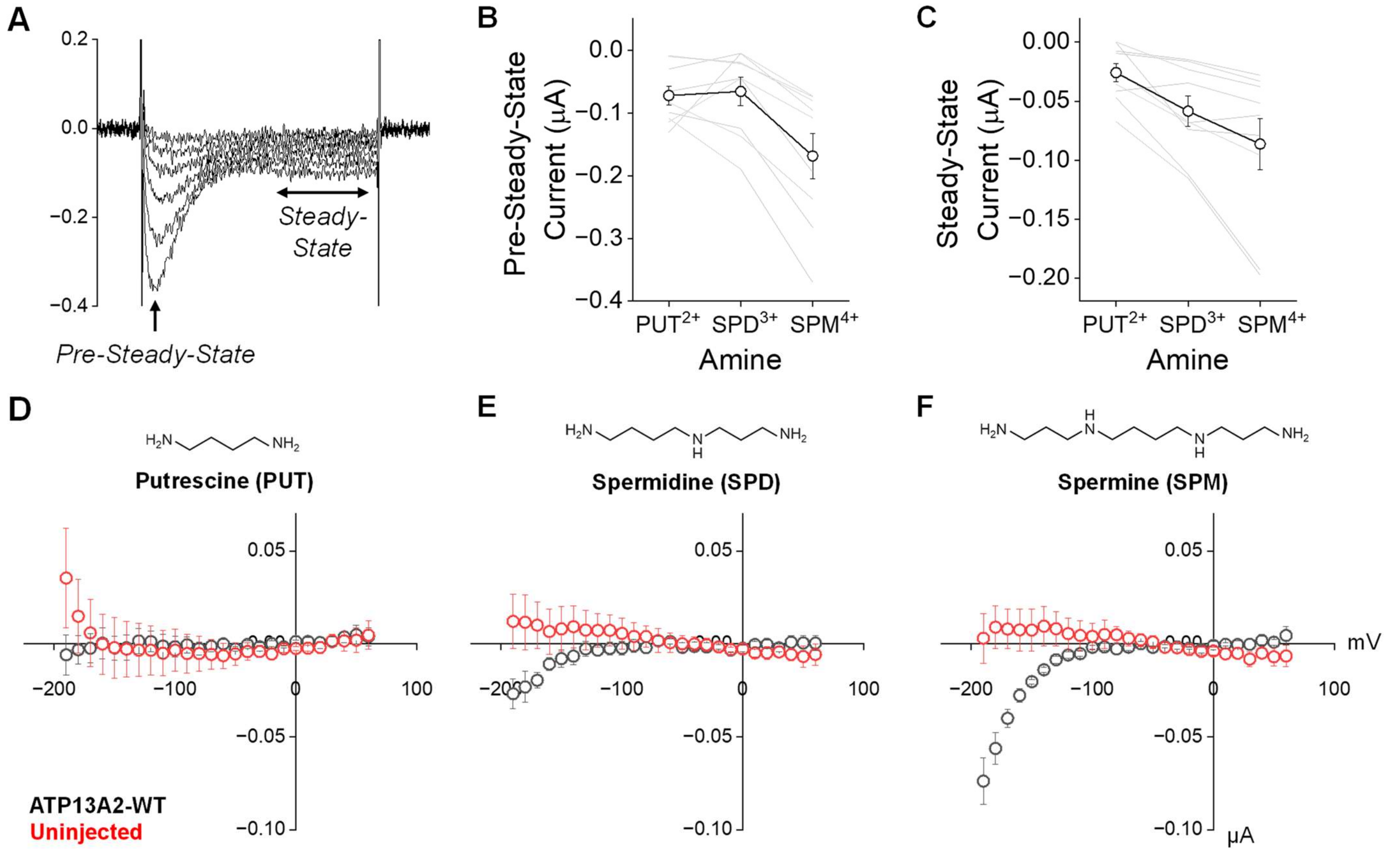

Supplementary Figure S6. A) Representative traces of currents elicited from ATP13A2 injected oocytes at -140 to -190 mV. **B, C)** Polyamine substrate-dependent activation pre-steady-state (*B*) and steady-state currents (*C*) ($n = 9$). **D-F)** Voltage-dependences of polyamine-induced currents activated by 1 mM PUT (*D*), SPD (*E*), and SPM (*F*) in oocytes injected with ATP13A2-WT cRNA (*black*, $n = 4$) or uninjected oocytes (*red*, $n = 3$).
