# Supplementary material for: Direct detection of electrogenic polyamine transport
Source: bioRxiv. 2026 Oct 7:2026.10.01.755726. Preprint. [Version 1] doi: 10.64898/2026.10.01.755726 (PMC13691954; doi:10.64898/2026.10.01.755726)
Supplement: Supplementary file 8 [file media-8.pdf]

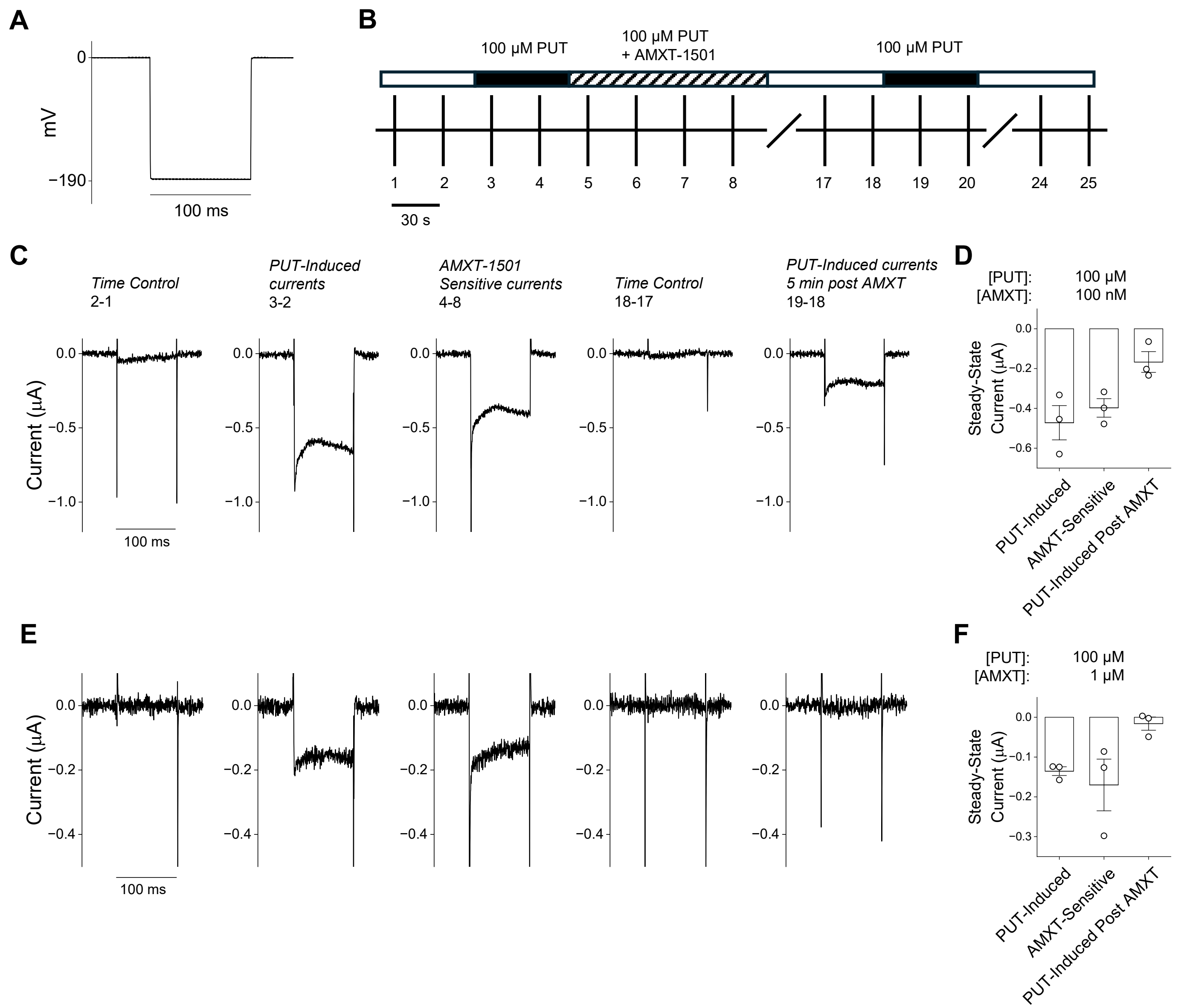

Supplementary Figure S8. A, B) -190 mV, 100 ms depolarizations (holding potential = 0 mV) were applied to ATP13A3-injected oocytes every 30 s while perfusing with bath solution or solution with 100 µM PUT ± a concentration of AMXT-1501. **C,D)** Only a fraction of the current induced by 100 µM PUT was blocked by 100 nM AMXT-1501. Some current was recovered after removing AMXT-1501 and perfusing with bath solution for 5 min. (*n* = 3). **E,F)** 1 µM AMXT-1501 completely abolished PUT-induced currents. PUT-induced currents were not recovered after removing AMXT-1501 and perfusing with bath solution for 5 min (*n* = 3).
