# Supplementary material for: Direct detection of electrogenic polyamine transport
Source: bioRxiv. 2026 Oct 7:2026.10.01.755726. Preprint. [Version 1] doi: 10.64898/2026.10.01.755726 (PMC13691954; doi:10.64898/2026.10.01.755726)
Supplement: Supplementary file 9 [file media-9.pdf]

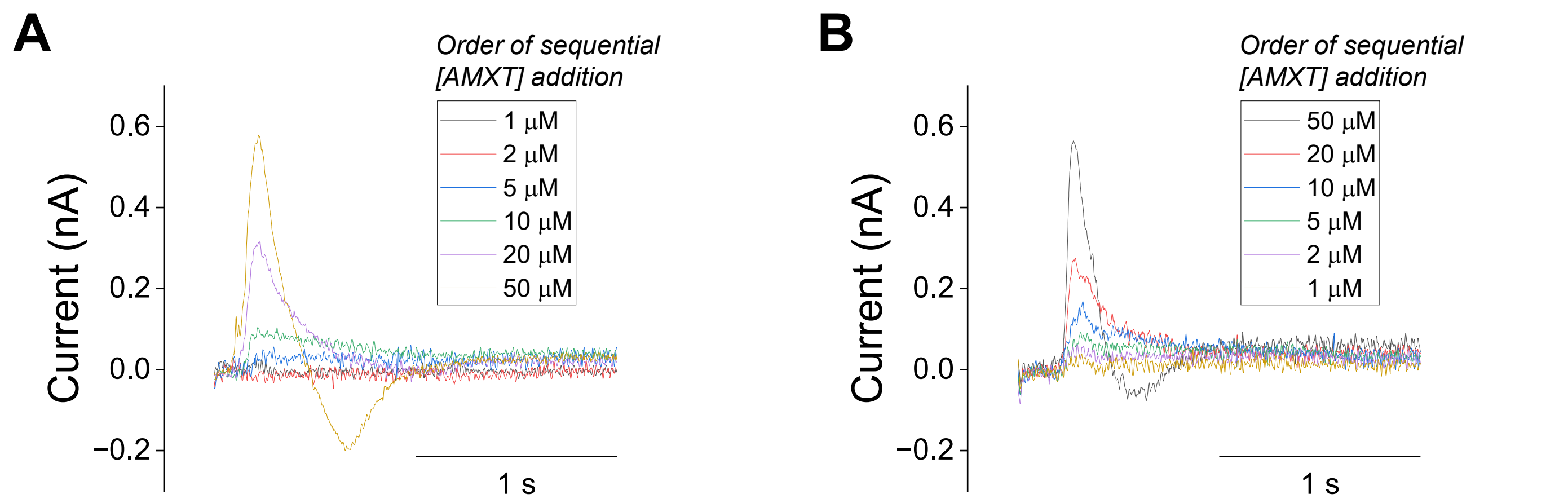

Supplementary Figure S9. A,B) Solid-supported membrane current traces were recorded following AMXT-1501 concentration jumps. The same concentration jumps were performed in ascending (*A*) and descending (*B*) AMXT-1501 concentration order, as indicated in the panels. Each trace was obtained by averaging five consecutive measurements ($n = 5$).
